# How advocacy groups on Twitter and media coverage can drive US firearm acquisition: A causal study

**DOI:** 10.1093/pnasnexus/pgaf195

**Published:** 2025-06-12

**Authors:** Kevin Slote, Kevin Daley, Rayan Succar, Roni Barak Ventura, Maurizio Porfiri, Igor Belykh

**Affiliations:** Department of Mathematics and Statistics, Georgia State University, P.O. Box 4110, Atlanta, Georgia 30302-410, USA; Department of Mathematics and Statistics, Georgia State University, P.O. Box 4110, Atlanta, Georgia 30302-410, USA; Center for Urban Science and Progress, Tandon School of Engineering, New York University, Brooklyn, NY 11201, USA; Department of Mechanical and Aerospace Engineering, Tandon School of Engineering, New York University, Brooklyn, NY 11201, USA; Center for Urban Science and Progress, Tandon School of Engineering, New York University, Brooklyn, NY 11201, USA; Department of Mechanical and Aerospace Engineering, Tandon School of Engineering, New York University, Brooklyn, NY 11201, USA; School of Applied Engineering and Technology, Newark College of Engineering, New Jersey Institute of Technology, Newark, NJ 07102, USA; Center for Urban Science and Progress, Tandon School of Engineering, New York University, Brooklyn, NY 11201, USA; Department of Mechanical and Aerospace Engineering, Tandon School of Engineering, New York University, Brooklyn, NY 11201, USA; Department of Biomedical Engineering, Tandon School of Engineering, New York University, Brooklyn, NY 11201, USA; Department of Mathematics and Statistics, Georgia State University, P.O. Box 4110, Atlanta, Georgia 30302-410, USA; Neuroscience Institute, Georgia State University, P.O. Box 4110, Atlanta, Georgia, 30302-410, USA

**Keywords:** causal inference, firearm acquisition, firearm violence, PCMCI+, social media

## Abstract

Firearm injuries are a leading cause of death in the United States, surpassing fatalities from motor vehicle crashes. Despite this significant public health risk, Americans continue to purchase firearms in large quantities. Commonly cited drivers of firearm acquisition include fear of violent crime, fear of mass shootings, and panic-buying. Additionally, advocacy groups’ activity on social media may capitalize on emotions like fear and influence firearm acquisition. The simultaneous effects of these variables have not been explored in a causal framework. In this study, we aim to elucidate the causal roles of media coverage of firearm laws and regulations, media coverage of mass shootings, media coverage of violent crimes, and the Twitter activity of anti- and proregulation advocacy groups in short-term firearm acquisition in the United States. We collect daily time series for these variables from 2012 to 2020 and employ the PCMCI+ framework to investigate the causal structures among them simultaneously. Our results indicate that the Twitter activity of antiregulation advocacy groups directly drives firearm acquisitions. We also find that media coverage of firearm laws and regulations and media coverage of violent crimes influence firearm acquisition. Although media coverage of mass shootings and online activity of proregulation organizations are potential drivers of firearm acquisition, in the short term, only the lobbying efforts of antiregulation organizations on social media and specific media coverage appear to influence individuals’ decisions to purchase firearms.

Significance Statement Understanding the drivers of firearm acquisition is essential for addressing firearm-related harms without infringing on citizens’ rights. Using a causal inference framework with a daily resolution, we show that short-term firearm purchases in the United States can be primarily influenced by the Twitter activity of antiregulation advocacy groups, media coverage of firearm laws, and media coverage of violent crimes. The results suggest that the activity of advocacy groups on social media and certain media narratives can directly impact firearm acquisition, offering valuable insights for policymakers and public health initiatives aimed at reducing firearm injuries.

## Introduction

Firearm violence is a major public health concern in the United States, where the incidence of deaths by firearms is steadily increasing. In the 20 years between 2001 and 2020, nearly 680,000 people died of firearm-related violence in the country (1). While in 2001, nearly 30,000 people died by firearms, by 2017, this figure reached almost 40,000, averaging 109 deaths per day and surmounting the number of deaths due to motor vehicle accidents (2). Firearm violence and injury have been strongly associated with accessibility to the agent causing the harm: firearms. Several studies showed that states with greater firearm ownership experience greater rates of suicides, homicides, and aggravated assaults with firearms (3–7). Despite the evidence of their harm, many Americans continue to purchase firearms in large amounts. Firearm ownership is central to American culture and identity and offers citizens a multitude of benefits, including physical activity, social interactions, stronger familial ties, and a connection to nature (8–11). Thus, to reconcile American citizens’ wishes to own firearms with the dire need to mitigate the risk of firearm harm, it is important to understand the factors driving firearm acquisition.

Perhaps the most cited driver of firearm acquisition is self-protection. A 2019 study by the Pew Research Center revealed that 67% of owners purchased firearms to protect themselves (12). Further, a poll conducted by NBC in 2018 showed that 58% of American adults believe that ubiquitous firearms would increase their communities’ safety by allowing law-abiding citizens to protect themselves (13). Research has attributed Americans’ need for self-protection in part to their fear of victimization in violent crimes (8, 14–19) and a 2016 Gallup survey showed that individuals who were victims of a crime are more likely to become firearm owners (20). However, research that elucidates whether fear of crime definitively translates to firearm acquisition is limited. Hauser and Kleck (21) analyzed surveys that followed up with respondents and were the first to show evidence that fear of crime materializes in firearm purchases. In the same study, Hauser and Kleck also found that the act of purchasing a firearm did not reduce the fear of violent crimes among nonowners, but the notion of relinquishing the weapon increased it.

Another path of victimization that deeply concerns Americans is fear of mass violence. The United States is unique for its rates of mass shootings, where more such events take place than anywhere else in the world (22, 23). Despite their relatively high prevalence in the United States, mass shootings over the 20 years between 1999 and 2019 accounted for only 0.36% of firearm homicides (1, 24, 25). Yet, the extensive media attention they garner leads people to believe they are more prevalent than they are in reality (26, 27). To this effect, surveys by The Harris Poll (administered on behalf of the American Psychological Association) indicated that 79% of Americans experience stress by the possibility of a mass shooting, and 33% of them do not attend public events out of fear of a mass shooting (28). Thus, mass shootings can induce “moral panic” (29–31), a perception of a threat that is disproportionately greater than the actual threat. Considering that mass shootings elicit an extraordinary sense of lack of personal safety among American citizens, Wallace suggested that mass shooting events lead to greater firearm acquisition (32). Supporting this proposition, empirical evidence by Refs. (33–38) shows that firearm sales spike following mass shootings.

Another possible driver of firearm acquisition in the United States is rooted in “panic-buying.” Panic-buying is a well-documented phenomenon where crowds anticipate future scarcity of a product and buy unusually large amounts of it (39). For example, introducing a “New Coke” beverage formula in 1985 led many consumers to panic-buy the original Coke until its depletion in stores (40). In the context of firearms, panic-buying would correspond to the rushed purchase of firearms and ammunition in anticipation of looming firearm regulation. Panic-buying of firearms was recorded in 2008 as demand for firearms surged following the election of President Barack Obama, whose political agenda included stricter firearm laws, in 2013 in New Jersey following Governor Christie’s proposal to expand background checks and ban rifles, and in Maryland before the ban of semiautomatic rifles (41, 42). Thus, it is expected that greater acquisition of firearms will be observed following the announcements of firearm regulations.

The public’s perception of potential victimization and their knowledge of upcoming firearm regulations are likely shaped by the portrayal of such events on mass media (43–46). As media capitalize on the attention they attract, outlets tend to sensationalize events to draw an audience. Prior studies have shown that news reports exaggerate stories of more violent crimes, and that the amount of media coverage of such events is disproportionate to their rate of occurrence (44). The media guide the public’s perception of social problems and even serve agendas (44, 47, 48), but also drive certain behaviors (46, 49). As such, media coverage of relevant topics could be a proxy of the public’s fear of violent crimes, mass shootings, and firearm restrictions.

The three aforementioned potential drivers of firearm acquisition were studied through the years in correlation studies, linear regressions, or evidence-based inferences. Recently, our group investigated their media coverage in a causal framework (50). We collected the count of mass shootings, the count of newspaper articles on shootings, the count of newspaper articles on firearm control, and the number of background checks (as a proxy for firearm purchases) in the country every month between 1999 and 2017. To quantify the interaction between each pair of the four variables, we computed transfer entropy between their time series. Transfer entropy assesses causality in a Wiener–Granger sense as the reduction in uncertainty of the prediction of the future of a variable, given knowledge about its history and the history of another variable (51). It can effectively quantify causal links in the presence of nonlinear interactions (where the interaction is dictated by a power-law or U-shaped relationship) and multiple time delays (52, 53), and also in instances where counterfactual measurements are absent (54). Among all pairwise interactions we inspected, our results indicated that only two links are causal: the one from mass shootings to media coverage of shootings and the one from media coverage of firearm control to background checks. These findings were further supported by subsequent analyses (55, 56).

The results raise two questions. First, it is possible that the links from mass shootings to background checks and from media coverage of shootings to background checks were not found because the process between those variables takes place in a shorter period of time than the monthly resolution of data. That is, people are exposed to breaking news on violent crimes and mass shootings and prompted to purchase firearms within days, not months. Second, it is tenable that replacing the time series of mass shootings with a time series of media coverage of mass shootings would better reflect the public’s perception of danger from these events. In the present study, we expand on the analysis in Porfiri et al. (50) and perform it with daily data so that we capture processes that take place on a shorter time scale of days. Such an analysis could not have been conducted before, as the Federal Bureau of Investigation (FBI) only made daily background check data available in 2021.

In addition to these innovations, this study offers insight into another potential driver of firearm acquisition: the activity of interest groups on social media. American interest groups exist for a wide range of societal, economic, and environmental causes, including public healthcare, abortion, immigration reforms, and climate change (57). There are two opposing interest groups in the domain of firearms: antiregulation organizations and proregulation organizations. Firearm enthusiasts contend that the US Constitution protects the right to own a firearm. In contrast, proponents of firearm control are often prompted by firearm-related tragedies to advance legislation that restricts access to firearms. Both interest groups are well-represented at the state and federal levels of government. With the rise of social media, Facebook, Twitter (now X), and YouTube have become substantial platforms for interest groups to express their agendas through “outside lobbying” (58). Outside lobbying refers to interest groups’ attempts to mobilize citizens rather than policymakers to influence public officials (58). Pro- and antiregulation organizations also use these platforms to promote their agendas among citizens; however, research on the topic suggests that these organizations adopt different strategies. Auger assessed the use of social media by pro- and antiregulation nonprofits and found that while both engaged with their communities and expressed gratitude toward their stakeholders, the former are more likely to emphasize conflict on their social media channels while the latter publish content about politics and legislation (59). In agreement with this finding, Merry showed that the timing of content release differs among the interest groups, whereby proregulation organizations’ activity peaks following mass shooting events and emphasizes victims and heroes (60). In contrast, antiregulation organizations published less content around these events and focused on legislative actions. In both cases, as interest groups aim to gain traction among their followers, they likely target identity issues and emotions (61).

The strategic use of social media by interest groups not only shapes the public’s perception of current events but also contributes to broader societal trends, such as political polarization. Studies have shown that social media platforms amplify partisan sorting, where individuals increasingly engage with extreme rather than moderate views, deepening divisions between opposing political groups (62). This process of partisan sorting is influenced by users’ tendency to form social ties based on shared partisanship (63). Furthermore, exposure to opposing views on social media can increase political polarization, further entrenching the opposing stances of interest groups (64). Studies also highlight that political segregation on social media is driven not only by homophily but by acrophily, a preference for engaging with more politically extreme users rather than moderate ones, which can amplify inter-group antagonism (65).

While the literature on the *modus operandi* of anti- and proregulation organizations is vast, studies on their influence on public behavior are limited, and causal analysis in this domain is scarce. This study is the first to analyze the role of both interest groups as potential drivers of firearm acquisition. Given that this study examines links between six variables that are likely interacting (firearm acquisition, activities of anti- and proregulation interest groups, and media coverage of firearm laws and regulation, mass shootings, and violent crime), transfer entropy is not an ideal approach for this endeavor due to the curse of dimensionality (66, 67). Thus, we employ Peter-Clark Momentary Conditional Independence (PCMCI)+, a framework for discovering links within a graph (68). Building on the PC algorithm (69), PCMCI+ was designed for high-dimensional data sets where it successfully captures contemporaneous interactions and delayed dependencies.

Here, we investigate the role of mass and social media in driving firearm acquisition within a time scale of days. We report results for PCMCI+ applied to five potential drivers of firearm acquisition: media reports on violent crimes, media reports on mass shootings, media reports on firearm regulations, and activities of both anti- and proregulation organizations on social media.

## Methods

We collected daily data about six variables, summarized in Table 1: (i) media coverage of firearm laws and regulation, (ii) media coverage of mass shootings, (iii) media coverage of violent crime, (iv) Twitter posts (colloquially known as “tweets”) by antiregulation organizations, (v) tweets by proregulation organizations, and (vi) background checks.

**Table 1.** A summary of the data collected in this study.

Variable	Source	Total counts
Media coverage of firearm laws and regulation	ProQuest	10.4k
Media coverage of mass shootings	ProQuest	18.3k
Media coverage of violent crime	ProQuest	20.5k
Tweets by antiregulation organizations	Twitter (X)	155.4k
Tweets by proregulation organizations	Twitter (X)	238.5k
Background checks	FBI NICS	192,170k

For the first three variables (i–iii), media coverage was measured as the number of newspaper articles published on a topic collected from the ProQuest search engine (access provided by Georgia State University libraries) (70). Numbers were obtained using a procedure identical to the one reported in Porfiri et al. (50). Beginning with media coverage of firearm laws and regulations (variable i), we searched for the term “firearm laws” and included “firearm laws and regulations” in the Subject filter. We set the Source Type to “Newspapers” and specified the following 10 Publication Titles: Arizona Republic, Chicago Tribune, Denver Post, Houston Chronicle, Los Angeles Times, New York Times, Orlando Sentinel, St. Louis Post-Dispatch, Times-Picayune, and Wall Street Journal. These 10 daily news outlets represent liberal and conservative readerships across the US regions (71, 72) and were selected to ensure both ideological and geographic diversity in our media sample. The set includes newspapers from the Northeast, South, Midwest, West, and Mountain West, thereby avoiding an overrepresentation of East Coast perspectives. While the sample size of 10 may appear limited, it reflects a balance between breadth and feasibility in a high-resolution, article-level content analysis. Including additional newspapers such as the New York Post, Washington Times, or Boston Globe may further increase geographic or ideological diversity but would also introduce redundancy with existing outlets that already capture similar political leanings and regional audiences.

To quantify media coverage of mass shootings (variable ii), we performed a similar search, querying newspaper articles about “shootings.” Within the Subject filter, we included “shootings” or “mass murder” and excluded “firearm laws and regulations.” Finally, we used the term “violent crime” when searching for media coverage of violent crime (variable iii). The same Source Type and Publication Titles were specified in both searches. The results for each query were exported to a comma-separated values (csv) document and aggregated by date to obtain the daily number of articles. The queries to reproduce these time series are available in Supplementary material Table 1. A Venn diagram in Supplementary material Fig. 1 shows that the time series of media coverage are largely independent and do not share a large proportion of articles among themselves.

To quantify the activity of pro- and antiregulation organizations on social media, we turned to Twitter (now X). Twitter is a popular micro-blogging platform where account holders can publish textual content with up to 280 characters, known as tweets. Twitter is commonly used by individuals and organizations (73), with affinity to firearms and otherwise (74, 75), who tend to post tweets in response to current events in real-time (76, 77). Until its recent rebranding as X, Twitter has allowed the scraping of tweets with highly granular time stamps and, therefore, was considered by social scientists as a “thermometer” of public discourse (78–80). To select the most influential organizations, we queried Social Bearing (81), an analytical tool dedicated to Twitter that stores the number of followers for each account. We identified the accounts that advocate for or against firearm regulation by searching for those that mention the keyword “gun” the most (a feature offered by Social Bearing). We sorted the returned accounts by follower count in descending order and visually inspected their Biographies. Our selection of accounts was limited to those corresponding to firearm control organizations. That is, we excluded accounts for individual persons, organizations that address other issues related to guns, and organizations that did not have official statements regarding firearm regulation. We then categorized the top accounts by stance (anti- or proregulation) based on the account biography, whether it was promoting or discouraging firearm regulation. We selected the most followed accounts for antiregulation organizations with over 5,000 subscribers (59, 82). Organizations like the National Rifle Association had multiple accounts, one nationwide and a few dedicated to local chapters. We selected the principal account with the largest geographical and social coverage if multiple accounts were returned. Overall, nine antiregulation accounts were included in the study. To mirror this selection, we picked the most-followed accounts of proregulation organizations, again thresholding at 5,000 followers, yielding 11 organizations. In total, 20 organizations were included in this study.

To generate a time series for each interest group’s activity on social media, we collected the number of posts each published. Specifically, we searched for posts containing the keyword “gun” by each organizational account using the “Counts” endpoint of the Twitter Academic Research API v2 (83). All Twitter data were collected between 2022 February 2 and February 26.

To measure the rates of firearm acquisition, the daily number of background checks performed across the United States was obtained from the FBI’s National Instant Criminal Background Check System (NICS) (84). Although background checks are not a direct measure of firearm purchases (85), they are commonly used as a proxy in the absence of a national registry (32, 50, 56, 86). For all variables, the time series began in January 2012 when Twitter saw a surge in registered users (87). To avoid anomalies related to the COVID-19 pandemic (88–90), the time series was truncated on 2020 January 1. As such, we generated six time series (one for each variable), each containing 2,923 daily counts between 2012 January 1 and 2020 January 1.

All time series were seasonally adjusted and detrended using the forecast package on R (version 8.15; (91)). Seasonal adjustment was applied for periods of 1,2,3,5,7,30,31, 365.25/12, and 365.25 days. Subsequently, the treated time series were tested for the presence of trends using both the Augmented Dickey–Fuller (ADF) and Kwiatkowski–Phillips–Schmidt–Shin (KPSS) tests (92, 93).

We performed causal analysis on the detrended and deseasonalized time series using PCMCI+ with the software package Tigramite (68). PCMCI+ begins with a complete graph *G*, where each node $X_{t_{n}}$ represents the time series of a variable at a certain time delay. The subscript n∈(1,2,…,N) corresponds to a variable, and the superscript t∈(T,T−1,…,T−τ) corresponds to a delay of the variable such that *N* is the total number of variables, *T* is the entire time series length, and τ≥0 is the maximum delay tested for in the algorithm. The algorithm considers all specified delays applied on all variables simultaneously, thereby controlling for autocorrelations within each time series (68).

The algorithm is based on a variant of the PC algorithm (69) and the concept of momentary conditional independence (MCI), inspired by the information-theoretic measure of momentary information transfer (94). The skeleton is discovered by heuristically testing pairwise independence between variables and later independence between variables conditioning on a set of the parents that are updated in each iteration of the algorithm. Once the skeleton of the time series graph convergences, links are oriented based on time delays for time dependencies, and based on deterministic rules for contemporaneous links; that is, if a link exists between $X_{t_{1}}^{i}$ and $X_{t_{2}}^{j}$, the orientation of the link between *i* and *j* is posed based on the difference between $t_{1}$ and $t_{2}$ (i→j if $t_{1}<t_{2}$ and j→i if $t_{2}<t_{1}$). For $t_{1}=t_{2}$, deterministic rules based on Pearl’s causality are applied to determine the orientation (95). Finally, during the momentary conditional independent phase, a link is established if and only if the variables are not independent, given the set of the parents of both the sink and the source variables from the skeleton graph.

When applying the PCMCI+ algorithm, we specified possible delays of τ=0,1,2,3,4,5,6, and 7 days, and designated partial correlation *ρ* as the measure of conditional dependence between pairs of variables. The sign of *ρ* was used to determine whether an association is positive (both variables increase/decrease together) or negative (one variable increases while the other decreases).

The PCMCI+ algorithm operates under standard causal assumptions: causal sufficiency, faithfulness, and the Markov condition. Faithfulness and the Markov condition are inherently integrated into the algorithm’s steps—removing links between statistically independent nodes while retaining significant dependencies. Causal sufficiency, on the other hand, assumes that no relevant, unobserved time-series variables influence the observed system. This means the accuracy of the method depends on incorporating all relevant variables based on domain knowledge. Once this assumption is met, PCMCI+ efficiently mitigates the curse of dimensionality by iteratively and optimally accounting for potential confounders. A key advantage of PCMCI+ over other casual discovery methods (96–98) is its ability to handle fast dynamics, effectively addressing both lagged and contemporaneous dependencies, including both directed and undirected contemporaneous links. However, the algorithm’s performance can be influenced by its hyperparameters. To ensure robustness, simple sensitivity analyses can confirm the stability of the results.

To assess the robustness of the causal links identified by the PCMCI+ algorithm, we conducted a link persistence analysis. Specifically, we ran the algorithm 30 times, systematically varying two key parameters: the maximum time lag *τ*, ranging from 5 to 14, and the significance threshold *α*, set at 0.05, 0.01, and 0.001. For each combination of *τ* and *α*, we recorded whether each link was found to be significant in the MCI step of PCMCI+. Link persistence was then defined as the proportion of simulations (out of 30) in which a given link appeared at the exact time lag.

To verify the existence of the causal links we found, we compared the results with the traditional causal method of Granger (96). The pairwise Granger causality test uses regression models to assess whether past values of a source variable improve the prediction of a sink variable. Two regression models were examined, one where the lagged values of the sink variable predict themselves in an autoregressive manner (known as the restricted model) and another one where the lagged values of both the source and sink variables predict the sink variable (the full model). Should the source variable Granger–Cause the sink variable, the residuals of the two regression models would be statistically different whereby the sum of residuals squared would be lower for the full model (99). Under the null hypothesis that the residuals of the models are not statistically different, the F-statistic was calculated to determine significance. We applied pairwise Granger causality with background checks as the sink variable and each of the other five variables as a source variable.

## Results

**Fig. 1.**
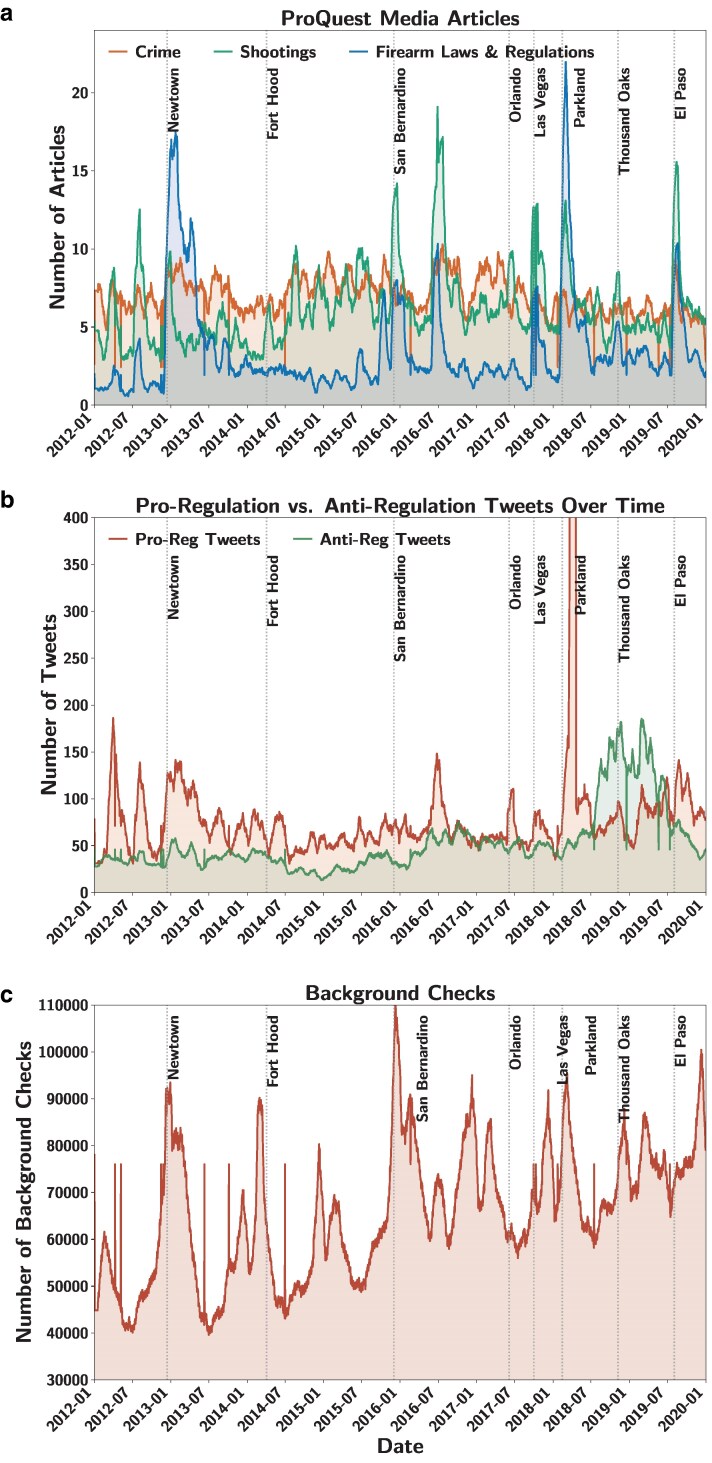
Daily time series of the variables considered in the study: a) media coverage of firearm laws and regulation, mass shootings, and violent crime, b) tweets by proregulation organizations and antiregulation organizations, and c) background checks. A 30-day moving average filter was applied on the time series for clarity. A maximum peak of 12,586 tweets by proregulation organizations was observed following the Parkland mass shooting, but truncated from the plot. Vertical lines correspond to the following deadly mass shootings (left to right): Sandy Hook (12/14/2012 in Newtown, CT), Fort Hood (4/2/2014 near Killeen, TX), Inland Regional Center (12/2/2015 in San Bernardino, CA), Pulse Nightclub (6/12/2016 in Orlando, FL), Route 91 Harvest Festival (10/1/2017 in Las Vegas, NV), Marjory Stoneman Douglas High School (2/14/2018 in Parkland, FL), Borderline Bar and Grill (11/7/2018 in Thousand Oaks, CA), and Walmart (8/3/2019 in El Paso, TX).

Complete time series were collected for all six variables (Fig. 1). Media coverage of firearm laws and regulations contained a total of 10,431 articles, with a notable activity following the Sandy Hook school shooting on 2012 December 12, reaching a peak of 51 articles in January a month later (Fig. 1a). Peaks were also observed following the San Bernardino, Orlando, Las Vegas, Parkland, and El Paso shootings. Media coverage of mass shootings resulted in a larger total of 18,338 articles, with a peak value of 53 articles observed on 2016 July 9, 27 days after the Orlando shooting (Fig. 1a). In this time series, multiple peaks could be associated with mass shootings: all those highlighted in blue in Fig. 1a, as well as the DC Navy Yard shooting on September 16, 2013, Isla Vista mass murder on 2015 May 23, and the Congressional Baseball Shooting on 2017 June 14. Finally, media coverage of violent crimes contained a total of 20,511 articles, with a peak of 27 articles recorded on 2016 September 30 (Fig. 1a). Peaks in this time series were not so clearly associated with major mass shooting events. A list of all the organizations used to count tweets is available in Table 2. Although antiregulation organizations enjoy greater followership (1.76 million subscribers vs. 1.32 million), proregulation organizations publish many more posts. Tweets by proregulation organizations included 238,545 posts, with 12,586 published on 2018 March 24, following the Parkland shooting (Fig. 1b). Tweets by antiregulation organizations included 155,417 posts, with many published in the months following the Parkland shooting and a peak of 490 published on 2018 September 18 (Fig. 1b). Finally, the time series of background checks exhibited significant weekly and monthly seasonality, whereby firearm sales surged in the periods surrounding the holiday season and dipped in the summer (Fig. 1c). In this time series, too, peaks were not clearly associated with major mass shooting events.

**Table 2.** A list of the most followed Twitter accounts of anti- and proregulation organizations, along with their respective counts of followers and Twitter posts containing the keyword “Gun.”

Category	Handle name	Organization name	Number of followers	Number of posts
Antiregulation	nra	National Rifle Association	917.2k	16,527
	gunowners	Gun Owners of America	333.5k	8,463
	gunpolicy	Firearms Policy Coalition	215.5k	12,519
	usacarry	USA Carry	86.6k	34,849
	natlgunrights	National Association for Gun Rights	64.8k	6,356
	uscca	US Concealed Carry Association	64.8k	49,746
	blkgunsmattr	Black Guns Matter	43.5k	4,279
	bearingarmscom	BearingArms.com	25.3k	22,238
	naaganational	National African American Gun Association	14.8k	405
Proregulation	amarch4ourlives	A March for our Lives	440.4k	25,866
	momsdemand	Moms Demand Action	343.6k	37,289
	everytown	Everytown USA	267.6k	21,739
	giffordscourage	Giffords	104.7k	17,326
	bradybuzz	Brady: United Against Gun Violence	76.8k	15,316
	newtownaction	Newtown Action Alliance	46.4k	21,562
	csgv	Coalition to Stop Gun Violence	36.8k	58,865
	protesteasyguns	Protest Easy Guns	8,797	24,462
	efsgv	Educational Fund to Stop Gun Violence	3,830	2,511
	gunsdownamerica	Guns Down America	15k	2,599
	wagv	Women Against Gun Violence	22.5k	10,951

**Fig. 2.**
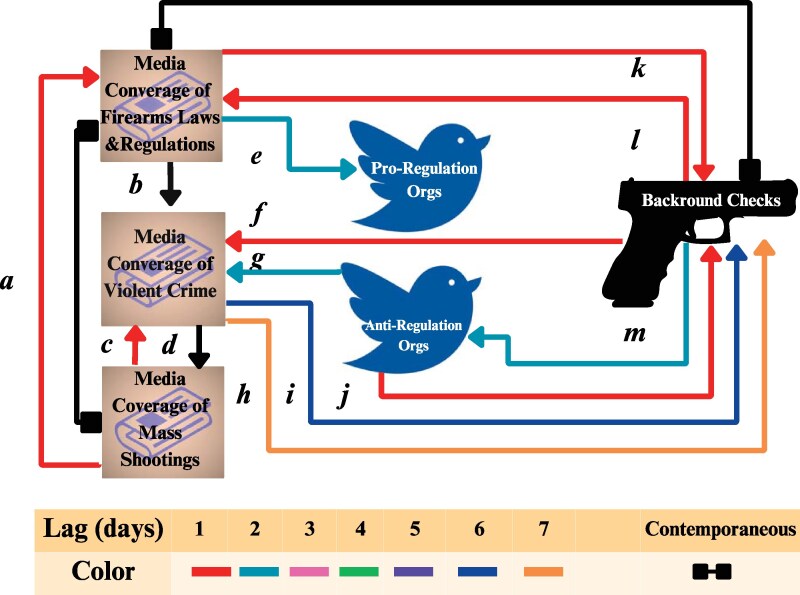
Causal diagram generated through PCMCI+ for the six variables under consideration. The colors of links reflect the time delay between the two variables they connect. Black links represent contemporaneous associations (0-day delay), with square endings indicating that the link is nonorientable. Red, teal, pink, green, purple, blue, and orange arrows reflect associations with delays of 1, 2, 3, 4, 5, 6, and 7 days, respectively. Links for delays of 3, 4, and 5 days were not recovered in the analysis.

All time series were appropriately detrended, reflected by *P*-values <0.05 for all ADF tests (where the alternative hypothesis is stationarity) and <0.1 for all KPSS tests (where the alternative hypothesis is nonstationarity; Table 3). Plots of processed time series are displayed in Supplementary material Fig. 2. PCMCI+ produced the graph in Fig. 2, with the associated quantities summarized in Table 4. Overall, 15 links were identified in the analysis: two unorientable contemporaneous links, two orientable contemporaneous links, and 10 orientable links.

**Table 3.** Results for ADF and KPSS stationarity tests performed on seasonally adjusted and detrended time series.

Variable	ADF *P*-value	KPSS *P*-value
Background checks	$3.703\times 10^{-3}$	>0.1
Media coverage of firearm laws and regulations	$2.092\times 10^{-5}$	>0.1
Media coverage of mass shootings	$2.439\times 10^{-22}$	>0.1
Media coverage of violent crime	$6.410\times 10^{-9}$	>0.1
Tweets by proregulation organization	$65.700\times 10^{-30}$	>0.1
Tweets by antiregulation organization	$1.041\times 10^{-1}$	>0.1

**Table 4.** Summary of the links identified by the PCMCI+ algorithm.

Link	Source variable	Sink variable	Delay (days)	*P*-value	Partial correlation coefficient *ρ*	Link persistence
a	Media coverage of mass shootings	Media coverage of firearm laws and regulation	1	<0.001	0.094	1.00
b	Media coverage of firearm laws and regulation	Media coverage of violent crime	0	<0.001	0.100	1.00
c	Media coverage of mass shootings	Media coverage of violent crime	1	0.001	0.083	1.00
d	Media coverage of violent crime	Media coverage of mass shootings	0	0.001	0.071	1.00
e	Media coverage of firearm laws and regulation	Tweets by proregulation organizations	2	<0.001	−0.069	1.00
f	Background checks	Media coverage of violent crime	1	<0.001	0.105	1.00
g	Tweets by antiregulation organizations	Media coverage of violent crime	2	0.009	0.048	0.57
h	Media coverage of violent crime	Background checks	7	<0.001	−0.075	0.40
i	Media coverage of violent crime	Background checks	6	0.035	0.054	0.33
j	Tweets by antiregulation organizations	Background checks	1	0.092	0.048	0.53
k	Media coverage of firearm laws and regulation	Background checks	1	<0.001	0.084	1.00
l	Background checks	Media coverage of firearm laws and regulation	1	<0.001	0.099	0.67
m	Background checks	Tweets by antiregulation organizations	2	0.001	0.059	0.97

The two unorientable contemporaneous links connected the node of media coverage of firearm laws and regulations with the nodes of media coverage of mass shootings and background checks. The two orientable contemporaneous, along with two noncontemporaneous links, entangled the nodes of media coverage. Link *a* extended from media coverage of mass shootings to media coverage of firearm laws and regulations, at a delay of 1 day (P<0.001). Contemporaneous link *b* was directed from media coverage of firearm laws and regulations to media coverage of violent crimes (P<0.001). Lastly, links *c* and *d* connected media coverage of mass shootings and media coverage of violent crime in opposing directions. While *c* represented a link from the former to the latter with a delay of 1 day (P=0.001), *d* represented a contemporaneous link from the latter to the former (P=0.001). All four links consisted of positive associations (ρ=0.094, 0.100, 0.083, and 0.071 for links *a*, *b*, *c*, and *d*, respectively).

Four links reflected the associations of tweets by interest groups. Tweets by proregulation organizations were only associated with media coverage of firearm laws and regulations (link *e*), where the interaction was directed from the latter to the former (P<0.001) and represented a negative association (ρ=−0.069). Tweets by antiregulation organizations were associated with media coverage of violent crime and background checks. One positive link (labeled *g*) was directed towards media coverage of violent crime with a 2-day delay (ρ=0.048; P<0.001). Two additional links indicated interactions with background checks. Link *j* represented a positive association, extending towards background checks with a delay of 1 day (ρ=0.048; P=0.092), whereas link *m* represented a negative association, extending away from background checks with a 2-day delay (ρ=0.059; P=0.001).

The remaining five associations connected background checks with different types of media coverage. Links *k* and *l* represented associations between background checks and media coverage of firearm laws, oriented in opposite directions (P<0.001). Both associations were positive (ρ=0.084 and 0.099, respectively) and consisted of a single-day delay. Links *f*, *h*, and *i* connected background checks with media coverage of violent crime with delays of 1, 7, and 6 days, respectively. One link, *h*, consisted of a negative association (ρ=−0.075), whereas the other two, *f* and *i*, indicated a positive association (ρ=0.105 and 0.054, respectively).

The node corresponding to background checks was most connected, with four links directed towards it, three links extending away from it, and one unorientable link. Both media coverage of firearm laws and regulations and media coverage of violent crimes had seven links. Media coverage of firearm laws and regulations had three outgoing, two incoming links, and two unorientable links, whereas media coverage of violent crimes had four links directed towards it and three links extending from it. Media coverage of mass shootings was associated with four links: two facing outwards, one facing inwards, and one unorientable. Finally, the activity of interest groups was the least connected nodes in the recovered network. Tweets by antiregulation organizations influenced two other nodes and were influenced by one, whereas tweets by proregulation organizations were influenced by a sole node only.

Results of the robustness analysis, which are summarized in the Link Persistence column of Table 4, revealed that links *a–f*, *k*, and *m* are highly perseverant, persisting across maximum time lags and significance thresholds. Links *g*, *j*, and *l* also emerged the majority of the time, appearing in 57, 53, and 67% of their respective simulations. More specifically, link *j* appeared 16 times out of 30, with two occurrences at α=0.05, four at α=0.01, and 10 at α=0.001. Links *h* and *i* were the least persistent, although they emerged in 33 and 40% of simulations.

Pairwise Granger causality offers backing to the existence of links, as shown in Table 5. In particular, significant links were found for all sources that led to background checks, except for tweets by proregulation organizations.

**Table 5.** Summary of the links identified in pairwise Granger causality analysis.

Source variable	Sink variable	Lag	*F*-test statistic	*p*-value
Media coverage of firearm laws and regulation	Background checks	1	49.75	0.000
Media coverage of firearm laws and regulation	Background checks	2	118.72	0.000
Media coverage of firearm laws and regulation	Background checks	3	72.35	0.000
Media coverage of firearm laws and regulation	Background checks	4	58.5	0.000
Media coverage of firearm laws and regulation	Background checks	5	44.76	0.000
Media coverage of firearm laws and regulation	Background checks	6	47.61	0.000
Media coverage of firearm laws and regulation	Background checks	7	14.69	0.000
Media coverage of mass shootings	Background checks	1	14.91	0.000
Media coverage of mass shootings	Background checks	2	15.05	0.000
Media coverage of mass shootings	Background checks	3	9.46	0.000
Media coverage of mass shootings	Background checks	4	14.46	0.000
Media coverage of mass shootings	Background checks	5	8.53	0.000
Media coverage of mass shootings	Background checks	6	10.66	0.000
Media coverage of mass shootings	Background checks	7	3.39	0.001
Media coverage of firearm laws and regulation	Background checks	1	86.02	0.000
Media coverage of firearm laws and regulation	Background checks	2	74.75	0.000
Media coverage of firearm laws and regulation	Background checks	3	51.4	0.000
Media coverage of firearm laws and regulation	Background checks	4	33.14	0.000
Media coverage of firearm laws and regulation	Background checks	5	24.89	0.000
Media coverage of firearm laws and regulation	Background checks	6	32.3	0.000
Media coverage of firearm laws and regulation	Background checks	7	13.18	0.000
Tweets by antiregulation organizations	Background checks	1	136.00	0.000
Tweets by antiregulation organizations	Background checks	2	53.53	0.000
Tweets by antiregulation organizations	Background checks	3	28.46	0.000
Tweets by antiregulation organizations	Background checks	4	17.90	0.000
Tweets by antiregulation organizations	Background checks	5	10.27	0.000
Tweets by antiregulation organizations	Background checks	6	8.76	0.000
Tweets by antiregulation organizations	Background checks	7	3.71	0.001

Reported *P*-values are rounded to the third decimal place.

## Discussion

Firearms are central to American culture and identity, yet firearm injuries are a leading cause of death in the United States. Understanding the drivers of firearm acquisition is an essential first step to comprehending and curbing firearm harms, without infringing on citizens’ right to bear arms. In this study, we performed causal analysis using the PCMCI+ framework (68) to elucidate the relationships between five potential drivers of firearm acquisition and firearm acquisition itself. This analysis extends on previous work from our group (50), showing that firearm acquisition in the United States is primarily driven by media coverage of firearm laws and regulations and media coverage of violent crime, but not by media coverage of mass shooting events.

Relative to Porfiri et al. (50), the innovations in the present study are three-fold. First, we investigate interactions that take place within days rather than months. Such an analysis with finer temporal resolution could reveal faster dynamics and unveil certain links that were previously absent (50). Second, we consider media coverage of mass shootings (rather than the incidence of mass shootings themselves) and media coverage of violent crimes. Research has shown that the amount of media coverage of criminal events is disproportionate to actual crime rates (95, 100) and that stories of criminal events are often sensationalized to capitalize on emotional narratives and draw more attention from the public (43, 101). Time series of media coverage of mass shootings and violent crimes may better correlate with people’s concern for their personal safety and tendency to purchase firearms rather than time series that directly measure the occurrence of those events. Third, we introduce two new nodes into the complex system that reflect the activities of anti- and proregulation organizations. The activity of both pro- and antiregulation organizations does not peak following mass shooting events, such that the time series possibly reflect ongoing public discourse about firearm regulation.

Our analysis revealed expected and unexpected results. The presence of contemporaneous links was not surprising. The time series of media coverage of mass shootings and media coverage of violent crimes encompass different aspects of violence: the former majorly refers to mass murder carried out with firearms. In contrast, the latter could refer to any crimes carried out with any means. However, the time series likely share some information whereby mass shootings can be considered a type of violent crime. As such, it is not surprising that when news about violent crime break, news about mass shootings break within the same day (link *d*). The direction of this association from the former to the latter could reflect preliminary reporting of a mass shooting event as a violent crime before details are fully unraveled and the event is deemed a mass shooting. Likewise, the contemporaneous link from media coverage of firearm laws and regulations to media coverage of violent crime (link *b*) was intuitive. It is reasonable that articles about firearm policies that allowed a perpetrator to obtain a weapon or could have prevented them from gaining access to weapons will co-occur with news about violent crimes with firearms. In this case, the direction of the association depicts the process whereby media initially report about legislative directions and later provide context of past violent crimes that could have been averted should a law had existed. This mechanism is independent of the nature of legislation as restrictive laws could prevent criminals from bearing firearms and permissive laws could allow victims to protect themselves. Relatedly, the co-occurrence of media coverage of mass shootings and media coverage of firearm laws and regulations was not surprising. Although mass shootings account for a small fraction of firearm deaths (102), they are very impactful in terms of public response: following these events, public discourse on firearms, firearm prevalence, and firearm regulation intensifies and prepares the ground for legislative action (103). Therefore, news about both variables could be released concurrently. Since the two topics are deeply intertwined, the presence of a link from media coverage of mass shootings to media coverage of firearm laws and regulations with a delay of 1 day (link *a*) is also highly conceivable. Finally, the unorientable contemporaneous link between background checks and media coverage of firearm laws and regulations could possibly demonstrate the causal link identified in Porfiri et al. (50), where people purchase firearms after they hear about upcoming regulations. This result suggests this behavior occurs within a single day.

In further agreement with Porfiri et al. (50), we found that background checks are influenced by media coverage of firearm laws and regulations (link *k*). Interestingly, we also found that background checks are influenced by media coverage of violent crimes (links *h* and *i*), indicating that many Americans purchase firearms for self-protection, although, they would act on this urge within the span of a week. These new two links, which were absent in previous studies with monthly time series (50, 55, 56), confirm our hypothesis that firearm acquisition in the United States is driven by slow and fast processes, and that analyses with finer temporal resolution could reveal faster dynamics.

Finally, our analysis revealed four links associated with the activity of interest groups on Twitter. Media coverage of firearm laws and regulations preceded Tweets by proregulation organizations by 2 days (link *e*); however, this association was found to be negative. Therefore, this link could reflect the efforts of those organizations to raise awareness towards legislation when it is not reported by the media. At the same time, our results did not support the notion that proregulation organizations capitalize on the occurrence of mass shooting events or violent crimes to promote their agenda (61). With respect to antiregulation organizations, we found that their activity on social media influences media coverage of violent crime (link *g*) with a delay of 2 days. Although antiregulation organizations publish less content following mass shootings and high-profile violent crimes (59, 61), their activity on social media might influence media narratives over time. In fact, the 2-day delay between antiregulation organizations’ tweets and increased media coverage of violent crime could be commensurate with the time needed for those organizations to gain attention from mass media on such platforms. By emphasizing legislative issues, antiregulation organizations may indirectly prompt media outlets to frame violent crime stories within policy debates on firearm regulations. This suggests their strategic focus contributes to shifts in media coverage after a short delay. The activity of antiregulation organizations on Twitter was also bidirectionally associated with background checks. Link *j* (from the former to the latter) is somewhat intuitive as it suggests that Twitter posts by antiregulation organizations, whose followership encompasses potential firearm buyers, elicit greater firearm acquisition. In contrast, link *m* (from the latter to the former) was unexpected. In addition to link *m*, our analysis points to two less expected links: from background checks to media coverage of violent crime (link *f*) and from background checks to media coverage of firearm laws and regulations (link *l*). We acknowledge that these links are not intuitively explained and may require additional analyses involving additional nodes in the graph.

In addition to the existence of causal links, we assessed their robustness. In agreement with previous studies (50, 55, 56), we found that media coverage of firearm laws and regulations consistently influence background checks, regardless of the lag and strictness of the significance threshold. Links among media coverage nodes were equally robust. Whilst the tweets by antiregulation organizations influenced background checks to a lesser extent, the link persisted in the majority of simulations, especially in situations where the conditional independence test was set to a higher threshold. It is possible that the link we recovered would have been more robust, had the activity of antiregulation advocacy groups included more prominent entities on social media such as gun influencers (also known as “gunfluencers” (104)). Should the means to reliably collect data on individual accounts become available, one could compare and contrast the role of influencers on firearm acquisition.

This study investigates the dynamics of mass media, the activity of interest groups on social media, and their influence on gun purchases. Nonetheless, our findings come with several limitations. In particular, while our study leverages high-frequency, large-scale data, we acknowledge that both outcome and input variables are subject to measurement limitations. First, the time series generated for this study may not fully reflect the public’s sentiment, discourse, or behavior. While background checks are commonly used as a proxy of firearm acquisition rates (32, 50, 56, 86), they fail to capture legal and illegal private party sales (32, 85). Alternative proxies of firearm ownership exist (7, 16); however, data on those variables are not available with daily resolution. Similarly, the time series of media coverage of firearm-related topics count new articles in 10 print media outlets. Although the selected outlets represent both liberal and conservative readerships across the US regions, the time series does not account for news reports from other means where people learn about current events, including television, radio, and the internet. Thus, inferring the public’s perception of crimes or knowledge of looming regulations from the time series may be inaccurate. Finally, the time series of tweets published by interest groups do not fully represent their activities and engagement with the public, as outreach could include interactive communication through comments and microvlogging, and in-person meetings (75, 105). This limitation comes as a cost of performing granular analysis with difficult-to-access daily data. Nonetheless, our results offer preliminary albeit circumspect insights into the drivers of firearm acquisition in the United States.

Although PCMCI+ is designed to be robust to random noise, systematic measurement error—especially if temporally correlated—may affect the estimation of dependencies and causal links. To mitigate these concerns, we used domain knowledge to inform keyword selection and conducted robustness checks across alternative lags and model settings. Nonetheless, we caution that these limitations may influence the precision of effect identification and encourage the interpretation of results in light of these data constraints.

Related to this limitation, one could argue that the use of national-level time series leads to the loss of state-level nuisances. Research has firmly indicated that firearm ownership varies among states, strongly affected by urbanization, demographics, socioeconomics, culture, and politics (8, 12, 19, 20, 56, 106, 107). Crimes rates and their nature also widely vary among states and regions (108, 109), and interest groups invest different levels of lobbying effort in each state (110). Ideally, the analysis presented herein would be conducted on a state level, however, daily data on background checks are not available nor is it feasible to collect geolocated data on media coverage of firearm-related topics and the activity of interest groups. Should such data become available, state-level analysis would be strongly warranted.

Second, the time series of media coverage originate in print media, whereas the time series of anti- and proregulation organizations come from social media. Print media is a slower means for publishing current events than digital media. That is, newspapers are distributed a day after events took place, whereas social media reflect events in real-time (80). This difference may have introduced a false delay in certain interactions. While news can be consumed through multiple fast-paced sources, tools that systematically record the content of news items in those media do not exist, and scraping such large-scale information from the internet is not feasible (111). An alternative approach to gauge public discourse might entail “infodemiology,” an emerging branch of science that queries determinants of information in digital media through social networks as well as search engines and other interfaces people engage with electronically (90, 112).

A third limitation in our study is the use of counts of Twitter posts without regard to their content. Future analysis could consider the sentiment and stance to quantitatively measure the kind of content anti- and proregulation organizations publish (89, 113). However, working with Twitter posts may provide limited information about the message organizations try to convey. In their study, Merry measured the “narrativity” of Twitter posts by the National Rifle Association and the Brady Campaign (60). For both organizations, they found that narrativity was lower than in other media and attributed this result to the platform’s limit of 140 characters. Further, although interest groups likely post content on all of their social media channels simultaneously, it is possible that including other outlets would have yielded different results as more narrated content is released to the public.

Finally, any evidence for contemporaneous links involving background checks or noncontemporaneous links that extend to/from background checks to other variables warrants cautious interpretation. Requests for background checks are submitted to NICS in a nonuniform manner and may not reflect the time a federally licensed firearm dealer submits a request accurately (85). When considering monthly time series, these uncertainties are mitigated to some extent. However, using daily data, we must consider that the delays associated with links are spurious.

## Conclusion

Overall, there are several take-home messages from this work. Concerning the temporal resolution of data, the results show a marked difference when comparing analyses with daily and monthly time series. Although daily studies offer a more immediate view, monthly analyses provide an encompassing perspective, which can be instrumental in discerning longer-term trends and subtler interconnections.

Regarding the drivers of firearm acquisition, while media coverage of violent crimes and firearm regulations may influence citizens’ choice to purchase a weapon, the activity of relevant interest groups also has direct implications on making this decision. Since candidate firearm owners are likely to subscribe to antiregulation channels, these organizations directly influence firearm acquisition, not proregulation organizations. With the understanding that media coverage of violent crime may drive firearm acquisition, as well as the activity of antiregulation organizations, legislators and policymakers are advised to target those aspects of the network to discourage firearm acquisition without limiting Americans’ right to bear arms. For example, media literacy initiatives could help people engage with emotionally charged news about violent crime critically, possibly reducing impulsive firearm purchases that are driven by fear. Further, given their ubiquity and importance, social media platforms could implement transparency measures regarding political advocacy campaigns, ensuring that content published by advocacy groups that is not based on sound scientific evidence is not disproportionately amplified through algorithmic biases. Finally, community-based violence prevention programs can provide alternative pathways to reduce violence and related crime. These targeted interventions could address key drivers of firearm acquisition while maintaining a balanced approach that respects constitutional rights.

## Supplementary Material

pgaf195_Supplementary_Data

## Data Availability

The data sets and codes used in the study are available on GitHub (https://github.com/Belykh-Lab/twitter-effect-supplemental).
