# Supplementary material for: How advocacy groups on Twitter and media coverage can drive US firearm acquisition: A causal study
Source: PNAS Nexus. 2025 Jun 12;4(6):pgaf195. doi: 10.1093/pnasnexus/pgaf195 (PMC12202873; doi:10.1093/pnasnexus/pgaf195)
Supplement: pgaf195_Supplementary_Data [file pgaf195_supplementary_data.pdf]

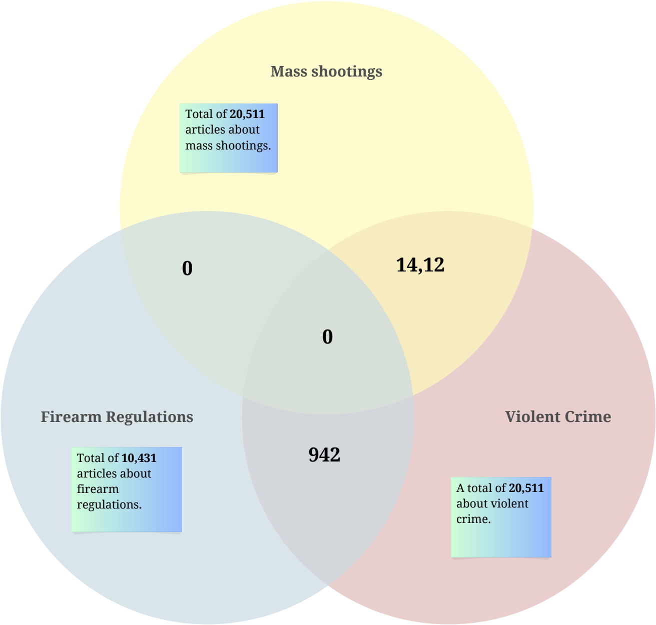

Supplementary Figure 1. Venn diagram showing the number of newspaper articles shared between the three time series of media coverage. More than 90% of the articles in each pair of time series are not shared.

Supplementary Information for “How advocacy groups on Twitter and media coverage can drive U.S. firearm acquisition: a causal study” by Kevin Slote, Kevin Daley, Rayan Succar, Roni Barak Ventura, Maurizio Porfiri, and Igor Belykh

Media Coverage of Firearm-Related Topics

We collected three time series from ProQuest, each containing the number of newspaper articles published on a firearm-related topic. The search queries used to generate the time series are listed in Supplementary Table 1. While the search terms for each topic were distinctly different, it is possible that the time series shared a substantial number of articles among them. For instance, the time series of media coverage of mass shootings and violent crimes could have shared a large number of articles, as mass shootings can be considered a type of violent crime. If this were the case, the time series generated would not be independent, and inference of causal relationships between them would be spurious.

In Supplementary Figure 1, we present a Venn diagram showing the number of newspaper articles shared between the time series of media coverage. While no articles are shared between media coverage of mass shootings and firearm laws and regulations, 942 articles are shared between media coverage of violent crimes and firearm laws and regulations, and 1412 between media coverage of violent crimes and mass shootings. In both cases, the number of shared articles represents less than 10% of the total counts in either time series. Therefore, the time series capture largely distinct processes.

Time Series Processing

The raw time series we gathered could contain seasonality and/or trends that could give rise to incorrect inference of interactions in an information-theoretic framework. Therefore, we seasonally adjusted and detrended the time series using the “forecast” package in R (version 8.18). Specifically, we applied the `msts` function for periods of 1, 2, 3, 5, 7, 30, 31, 365.25/12, and 365.25 days, followed by the `mstl` function to decompose the time series into seasonal, trend, cycle, and irregular components. We then applied the augmented Dickey–Fuller test to ensure the stationarity of the seasonally-adjusted and detrended time series. Supplementary Figure 2 displays the processed time series for each of the six variables.

Time Series	ProQuest Search Query
Media coverage<br>of firearm laws and regulations	(firearms laws)<br>AND (publication.exact("New York Times"<br>OR "Los Angeles Times"<br>OR "Chicago Tribune"<br>OR "Orlando Sentinel"<br>OR "St. Louis Post - Dispatch"<br>OR "Wall Street Journal"<br>OR "Arizona Republic"<br>OR "Denver Post"<br>OR "Times - Picayune"<br>OR "Houston Chronicle")<br>AND stype.exact("Newspapers")<br>AND subt.exact("firearm laws & regulations")<br>AND pd(20120101-20200101))
Media coverage<br>of mass shootings	(Shootings) AND<br>(publication.exact("New York Times"<br>OR "Los Angeles Times"<br>OR "Chicago Tribune"<br>OR "Orlando Sentinel"<br>OR "St. Louis Post - Dispatch"<br>OR "Wall Street Journal"<br>OR "Arizona Republic"<br>OR "Denver Post" OR "Times - Picayune"<br>OR "Houston Chronicle")<br>AND stype.exact("Newspapers")<br>AND subt.exact(("shootings"<br>OR "mass murders")<br>NOT "firearm laws & regulations")<br>AND pd(20120101-20200101))
Media coverage<br>of violent crimes	(violent crime)<br>AND (publication.exact("New York Times"<br>OR "Los Angeles Times"<br>OR "Chicago Tribune"<br>OR "Orlando Sentinel"<br>OR "St. Louis Post - Dispatch"<br>OR "Wall Street Journal"<br>OR "Arizona Republic"<br>OR "Denver Post"<br>OR "Times - Picayune"<br>OR "Houston Chronicle")<br>AND stype.exact("Newspapers")<br>AND pd(20120101-20200101))

Supplementary Table 1. ProQuest search queries used to collect the daily number of newspaper articles on firearm-related topics.

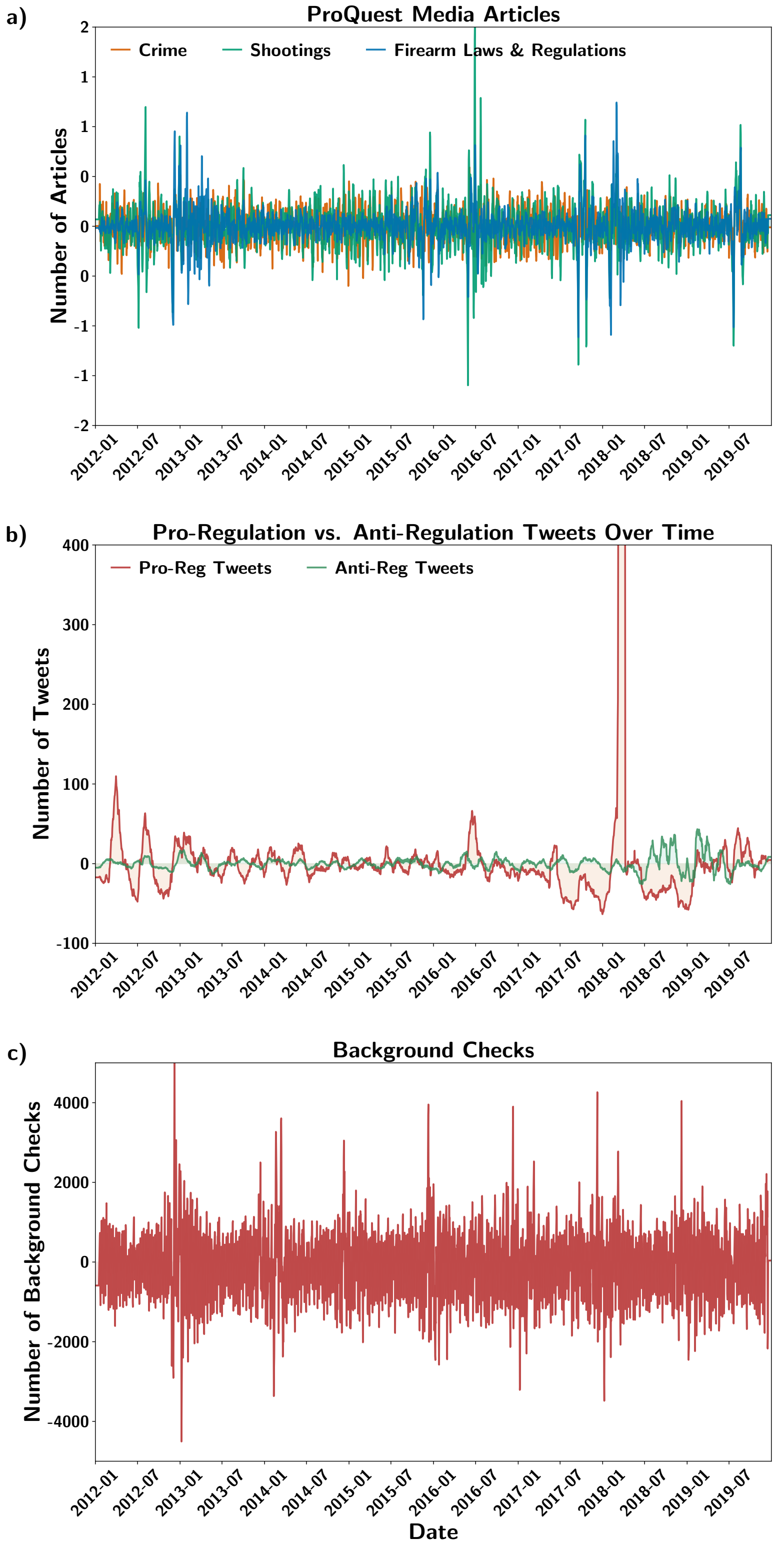

Supplementary Figure 2. Seasonally adjusted and detrended time series of the variables considered in the study: (a) media coverage of firearm laws and regulation (blue), mass shootings (green), and violent crime (orange), (b) tweets by pro-regulation organizations (red) and anti-regulation organizations (green), and (c) background checks. A 30-day moving average filter was applied on the time series for clarity.
